# Convention of Medical Teachers

**Published:** 1867-06

**Authors:** 


### CONVENTION OF MEDICAL TEACHERS.

A FEW days preceding the meeting of the "American Medical Association," representatives of nearly all the medical schools in America assembled to consult in regard to the various interests of medical education. This is a very important movement. A longer course, a more thorough curriculum, the preliminary qualifications of students, lecture fees, and a number of other things needed consideration, and we are gratified to learn that there was no sign of retrograding, but the meeting appeared to be alive to the demands of the age, and the interests of professional science. We are not fully informed as to all the good things likely to result from the meeting, and hence may refer to the matter more fully in a future number. The various Dental

Colleges have formed an Association, adopted a uniform curriculum, lengthened their sessions, and taken many other important steps likely to increase their usefulness. This subsequent action of the medical faculties will give their effort great moral support. W.

